# Narrative Review of the Role of Reactive Oxygen Species in Allergic Rhinitis

**DOI:** 10.3390/cimb48090947

**Published:** 2026-09-16

**Authors:** Jeongmin Lee, Su Young Jung, Hye Ok Kim, Jae Min Lee, Manish Kumar Singh, Sung Soo Kim, Jeon Gang Doo, Seung Geun Yeo

**Affiliations:** 1 Department of Medicine, College of Medicine, Kyung Hee University, Seoul 02447, Republic of Korea; sallyljm16@gmail.com (J.L.); hyeokkim@khu.ac.kr (H.O.K.); 2 Department of Otorhinolaryngology-Head and Neck Surgery, Myongji Hospital, College of Medicine, Hanyang University, Goyang 10408, Republic of Korea; monkiwh35@hanmail.net; 3 Clinical Research Institute, Kyung Hee University Medical Center, Seoul 02447, Republic of Korea; 4 Department of Otorhinolaryngology-Head and Neck Surgery, College of Medicine, Kyung Hee University Medical Center, Kyung Hee University, Seoul 02447, Republic of Korea; jmlee3042@khu.ac.kr; 5 Department of Biochemistry and Molecular Biology, College of Medicine, Kyung Hee University, Seoul 02447, Republic of Koreasgskim@khu.ac.kr (S.S.K.); 6 Department of Otorhinolaryngology-Head & Neck Surgery, Nowon Eulji Medical Center, School of Medicine, Eulji University, Seoul 02447, Republic of Korea

**Keywords:** allergic rhinitis, reactive oxygen species, oxidative stress, epithelial barrier, mitochondrial ROS

## Abstract

Studies of allergic rhinitis (AR) have increasingly recognized that reactive oxygen species (ROS) are not simply byproducts of oxidative metabolism but function as modulators of the type 2 inflammatory network during the pathogenesis of this disease. This narrative review summarizes the role of ROS in the pathophysiology of AR by analyzing studies that examined markers of systemic oxidative stress, dysfunction of the epithelial barrier, ROS derived from immune cells, mitochondrial redox signaling, and inflammasome-related pathways. Our structured search of the literature reviewed five major databases (PubMed, Scopus, EMBASE, Cochrane Library, and Google Scholar) and identified 17 eligible studies published between 2000 and 2026. Clinical studies suggest that patients with AR exhibit altered systemic redox homeostasis, including thiol–disulfide imbalance and increased lipid peroxidation. However, these findings are primarily from measurements of markers in peripheral blood, not nasal mucosa. Experimental studies consistently demonstrated that allergen exposure increased the levels of ROS in nasal epithelial and immune cells, disrupted the epithelial barrier, downregulated tight junction proteins, and activated inflammatory signaling pathways. Although direct nasal tissue data remain limited, evidence extrapolated from peripheral blood and bronchial challenge models suggests that eosinophils and neutrophils contribute to the generation of ROS during the late phase of the allergic response, thereby potentially amplifying and sustaining airway inflammation. There is also evidence that mitochondrial ROS and DUOX-dependent signaling contribute to epithelial dysfunction, including the release of damage-associated molecular patterns and activation of inflammasome pathways. In parallel, antioxidant defense mechanisms, such as the KEAP1/NRF2 axis and mitophagy-related pathways, appear to modulate disease severity by maintaining redox homeostasis. Experimental strategies such as ROS scavengers and oxidative stress-responsive drug delivery systems have shown early proof-of-concept potential in preclinical and pilot studies, but rigorous and large-scale clinical support is strictly required before any clinical application can be considered. Overall, current evidence indicates that ROS function in AR as context-dependent redox mediators rather than as primary causes. The biological effects of ROS appear to depend on site of synthesis, subcellular localization, and the balance between oxidant generation and antioxidant defenses. Further studies that directly assess the dynamics of nasal mucosal ROS and well-designed clinical trials are needed to clarify the translational relevance of these studies and the therapeutic potential of different treatments for AR.

## 1. Introduction

### 1.1. Allergic Rhinitis

Allergic rhinitis (AR) is a chronic inflammatory and systemic disease that occurs in sensitized individuals after inhalation of allergens that is due to an IgE-mediated type I hypersensitivity reaction. The clinical characteristics of AR include sneezing, watery rhinorrhea, nasal obstruction, and nasal itching. Some patients also experience rhinoconjunctivitis, with ocular symptoms such as itchy eyes, tearing, and conjunctival hyperemia.

The prevalence of AR depends on the specific diagnostic criteria, and also varies among populations, geographic regions, and age, but it is one of the most common upper airway diseases worldwide. AR adversely affects overall quality of life, including quality of sleep, academic performance, and workplace productivity [1,2,3]. The Allergic Rhinitis and its Impact on Asthma (ARIA) guideline and the International Consensus Statement on Allergy and Rhinology: Allergic Rhinitis 2023 (ICAR-Allergic Rhinitis 2023) emphasize that AR can occur with various airway and atopic diseases, including asthma, conjunctivitis, sinusitis, and otitis media. These comorbidities are a functional burden for individuals and a socioeconomic burden for societies [1,2]. However, AR differs among patients, in that there is considerable heterogeneity in phenotype, pattern of sensitization, causative antigen(s), and disease severity. The pathophysiology of AR consists of an inflammatory cascade induced by re-exposure to an allergen after sensitization. During the sensitization phase, the nasal epithelial barrier and mucosal immune system recognize inhaled allergens, and these are presented to naïve CD4+ T cells through antigen-presenting cells, especially dendritic cells. This is followed by a type-2 immune response induced by interleukin 4 (IL-4) and IL-13, which promotes the production of allergen-specific IgE, and the IL-5-mediated differentiation and survival of eosinophils. The IgE generated through this process binds to Fc epsilon RI (FcεRI) on the surface of mast cells and basophils. Upon re-exposure to the same allergen, IgE cross-linking rapidly induces the release of inflammatory mediators, such as histamine, leukotrienes, and prostaglandins [3,4,5]. This pathogenic process is generally divided into an early phase and a late phase. The early phase occurs immediately after allergen exposure and is characterized by sneezing, itching, and watery rhinorrhea, symptoms in which mast cell degranulation and histamine-mediated neural and vascular responses play central roles. The late phase develops several hours later and is characterized by persistent inflammation and nasal obstruction due to the recruitment of eosinophils, neutrophils, basophils, monocytes, and T cells into the nasal mucosa [3]. AR should therefore no simply be considered as an IgE-mediated immediate hypersensitivity reaction, but instead as a chronic inflammatory disease in which there is a combination of epithelial barrier abnormalities, innate immune activation, and type 2 cytokine signaling [2,3].

Recent studies have greatly increased our understanding of the role of the nasal epithelium in AR. The nasal epithelium is now regarded not merely as a physical barrier, but as a functional interface that actively regulates immune responses. Under normal conditions, the nasal epithelium serves as the first line of defense by blocking the penetration of external stimuli through tight junctions and mucociliary clearance. However, this barrier is not a fixed structure. Allergen-derived proteases, type 2 cytokines, and environmental pollutants can impair the integrity of the nasal epithelium, leading to increased penetration of allergens and the secretion of epithelium-derived inflammatory mediators [2,3]. These changes indicate that epithelial dysfunction is not merely a consequence of AR, but is a key component of the pathophysiology of AR.

### 1.2. Reactive Oxygen Species and Oxidative Stress

Reactive oxygen species (ROS) are highly reactive oxidizing molecules derived from molecular oxygen that include superoxide anion (O_2_•^−^), hydrogen peroxide (H_2_O_2_), hydroxyl radical (•OH), and lipid peroxides. These molecules differ in reactivity, half-life, range of diffusion, and biological targets, so studies of the biological effects of ROS must distinguish among these different species [6,7]. Earlier studies primarily regarded ROS as harmful byproducts that caused cellular damage, but more recent studies have emphasized that ROS also function as regulatory factors that function in physiological signaling. In particular, low concentrations of hydrogen peroxide can alter protein function, localization, and intermolecular interactions through the reversible oxidation of cysteine residues, and these processes are linked to the regulation of cell proliferation, differentiation, migration, metabolism, and stress responses [6,8]. This is an example of oxidative eustress—‘beneficial stress’.

Harmful oxidative stress occurs when the production of ROS exceeds the capacity of the antioxidant defense system or when ROS accumulate excessively in specific organelles. In this situation, the abnormal signaling can lead to the damage of proteins, lipids, and nucleic acids [6,7,8]. ROS are generated in various organelles, such as the mitochondria, endoplasmic reticulum, and peroxisomes, by enzymes including NADPH oxidase (NOX), dual oxidase (DUOX), xanthine oxidase, and uncoupled nitric oxide synthase. Mitochondria and the enzymes in the NOX/DUOX family are key axes that link the generation of ROS with inflammatory signaling in the respiratory epithelium and immune cells [6,7]. Moreover, the biological effects of ROS are not determined simply by the total level of ROS. Instead, their effects depend on the type of ROS, the organelle and enzymes responsible for their generation, their location within the cell, and the status of the surrounding antioxidant system. Therefore, the expression ‘increased ROS’ is generally considered insufficient [6].

Cells have various antioxidant defense mechanisms that help to maintain the redox balance, including superoxide dismutase (SOD), catalase, glutathione peroxidase, peroxiredoxin, and the thioredoxin system. In particular, the KEAP1/NRF2 system is a major adaptive response to oxidative stress. In this system, the oxidation of cysteine residues in KEAP1 leads to activation of NRF2, which then enters the nucleus and induces the transcription of several antioxidant genes, such as *HO-1*, *NQO1*, and *SOD* [5,7]. However, these antioxidant responses do not always have protective effects, and their effects can depend on the stage of disease, tissue, and source of the ROS [5,7]. The current evidence suggests that oxidative stress in AR should not be viewed as an initiating factor, but rather as a factor that modulates or amplifies inflammatory responses, epithelial dysfunction, mucus secretion, and immune cell activation within the context of IgE and type 2 inflammation.

Previous studies of patients with AR and experimental models have reported changes in biomarkers of oxidative stress, such as malondialdehyde (MDA), SOD, catalase, and glutathione, and suggested that AR may be associated with changes in NRF2, NF-κB, and epithelial barrier-related pathways [5]. However, measurement of these biomarkers alone is insufficient to conclude that an increased level of ROS in the nasal mucosa is the cause of AR.

Recent mechanistic studies have suggested several specific links between ROS and epithelial inflammatory responses. For example, there is evidence that stimulation of Th2 cytokines increases DUOX2-dependent generation of ROS in human nasal epithelial cells, and that these ROS may induce translocation of High Mobility Group Box 1 (HMGB1) and promote inflammatory responses [9]. These findings suggest that ROS are not merely oxidative byproducts, but may function as key signaling nodes that link dysfunction of the epithelial barrier, signaling by Damage-Associated Molecular Patterns (DAMPs), activation of inflammasomes, and mitochondrial dysfunction. However, most of the current evidence is based on in vitro studies, animal models, and a small number of clinical studies. The direct evidence of these effects in the human nasal mucosa remains limited.

Therefore, this review aims to characterize the roles of oxidative stress in AR by comprehensively examining the mechanisms of ROS generation; the changes in epithelial cells, immune cells, and barrier function during the early and late phases of disease; the generation of ROS by mitochondria; and the role of antioxidant pathways and therapeutic strategies that target ROS or the cellular responses to ROS. This narrative review therefore provides a balanced summary of the extent to which ROS act as regulatory factors in the pathophysiology of AR and of recent progress in this field.

## 2. Methods

Many studies have investigated the role of oxidative stress in airway diseases, but there has been no structured review that focused specifically on the molecular mechanisms of ROS in different phenotypes of AR. Therefore, a structured search of English-language publications was used to identify studies published between January 2000 and March 2026 in five electronic databases: PubMed, SCOPUS, Cochrane Library, EMBASE, and Google Scholar. The search strategy used combinations of the keywords “allergic rhinitis”, “reactive oxygen species”, “ROS”, and “mitochondrial ROS” with Boolean operators (“AND”, “OR”). To mitigate the risk of missing relevant studies indexed under related terms (e.g., oxidative stress, DUOX, SOD) that did not explicitly use the term ROS, this primary database query was supplemented by manual cross-referencing of the reference lists from retrieved articles and relevant previous reviews. Regarding species criteria, studies utilizing human subjects, patient-derived primary cells, and mammalian in vivo models (e.g., murine and guinea pig) were included to capture a comprehensive mechanistic overview, whereas non-mammalian models were excluded.

All included studies were original research articles; investigated ROS or free radical-related mechanisms in AR; and employed in vitro molecular assays, in vivo animal models, or analysis of human tissues. Studies were excluded if they were unrelated to AR; lacked mechanistic or biochemical data regarding AR; were review articles; or did not directly address redox signaling or oxidant/antioxidant imbalance.

**Figure 1:**
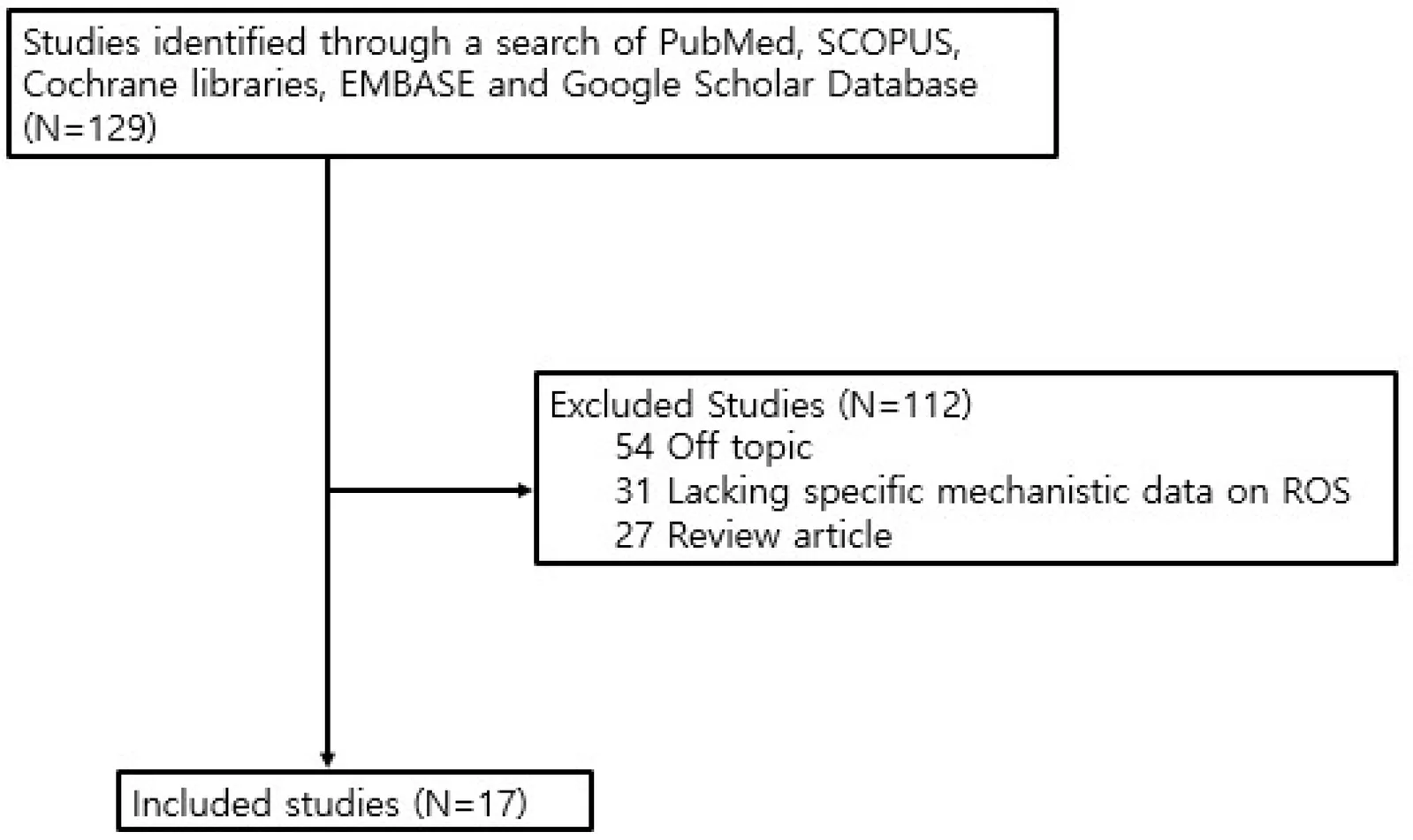
Procedure used to search the literature.

A total of 129 potentially eligible studies were initially identified. Then, 54 studies that were off-topic, 31 that lacked specific mechanistic data on ROS, and 27 review articles were excluded. Ultimately, 17 primary research articles were included for the qualitative analysis and synthesis. The selection process and transparent workflow was designed to minimize selection bias while maintaining the thematic focus of this narrative synthesis. Although a structured search approach was utilized to minimize selection bias, this study was designed as a narrative synthesis and does not strictly follow formal systematic review guidelines (e.g., PRISMA). Figure 1 shows the flow diagram of the study selection process.

## 3. Discussion

Table 1 summarizes the studies on ROS in AR that were reviewed in this article, including their study designs, samples, methods, target substances, results, and conclusions.

### 3.1. Clinical Relevance of Oxidative Stress in AR

AR is an upper airway type 2 inflammatory disease in which there is dysfunction of the epithelial barrier and an overactive immune response. ROS and oxidative stress regulate different molecules or pathways that may amplify inflammatory signaling in AR, with potential associations to disease severity and symptom burden [5]. However, most clinical evidence relies on measurements of biomarkers of oxidative stress in the systemic circulation rather than direct measurements of ROS in the nasal mucosa. Therefore, it is uncertain whether the oxidative imbalance in AR is a cause of the disease, a consequence of the inflammatory response, or simply an epiphenomenon. This section therefore summarizes the current clinical evidence about the role of ROS in AR by focusing on studies of biomarkers of systemic oxidative stress and environmental exposure to ROS in AR, and then discusses the limitations regarding the interpretations of these studies.

#### 3.1.1. Biomarkers of Systemic Oxidative Stress

Most studies of the clinical relevance of oxidative stress in AR have used indirect measurements of blood biomarkers. Overall, these studies suggest altered redox homeostasis in patients with AR, but this interpretation may be considered tentative because it is not based on direct measurements of ROS. Ulusoy et al. conducted a prospective case–control study comparing systemic oxidative stress during periods of symptomatic exacerbation and asymptomatic periods in patients with seasonal allergic rhinitis (SAR). This study included 32 patients with SAR and 32 age- and sex-matched healthy controls. The authors measured blood levels of native thiol (SH), total thiol (TT), disulfide (SS), and ratios of these molecules using a thiol–disulfide homeostasis assay. During periods of symptomatic exacerbation of SAR, the patients had a decreased level of native SH (347.7 ± 49.2 vs. 355.5 ± 53.5 μmol/L, *p* = 0.018) and an increased level of SS (23.4 ± 5.2 vs. 19.4 ± 5.8 μmol/L *p* = 0.014), but a similar level of TT (*p* = 0.212). In addition, the SS/SH and SS/TT ratios increased, and the SH/TT ratio decreased during symptomatic exacerbation, suggesting an increased oxidative stress (*p* = 0.001). Similar directional differences were also observed when compared with healthy controls. The authors concluded that symptomatic exacerbation of SAR is associated with an oxidative shift in the thiol–disulfide balance and that disease activity may reflect an increased systemic oxidative stress [10].

Another study of pediatric patients with perennial AR reported similar findings. Sadowska-Woda et al. measured erythrocyte SOD and catalase activity, MDA, plasma hydroperoxides, and ferric reducing antioxidant power (FRAP) in 50 children aged 3 to 10 years who had moderate perennial AR and in 11 healthy controls. Compared with the controls, the AR group had increased levels of lipid peroxidation markers and decreased activity of antioxidant enzymes (*p* ≤ 0.001). Administration of an antihistamine (desloratadine) led to some decrease in the level of hydroperoxides (but not complete normalization) and a partial improvement in antioxidant status. These findings suggest that oxidative stress occurs in pediatric patients with perennial AR and that an antihistamine may have some antioxidant effects beyond its blockage of histamine receptors [11].

#### 3.1.2. Environmental Exposure to ROS and Risk of Allergic Disease

However, not all studies have reported an association between oxidative stress and AR. To et al. analyzed the relationship between exposure to exogenous ROS during early life and the risk of developing allergic disease in a birth cohort. These researchers estimated the ROS burden generated by transition metals (Fe, Cu) in PM2.5 using air pollution data and a land-use regression model, and converted these data into individual exposures based on residential address at birth. The results indicated that early-life exposure to ROS had a significant association with the risk of childhood asthma (HR: 1.11, 95% CI: 1.02–1.21) but no significant association with AR (HR: 0.96, 95% CI: 0.88–1.04) or eczema. In addition, exposure to Fe alone or Cu alone was not associated with the risk of AR. These authors concluded that exposure to ROS may contribute to the development of asthma, but not AR [12].

#### 3.1.3. Interpretation of Current Clinical Evidence

Taken together, many recent clinical studies have reported increased oxidative stress-related changes in patients with AR, including an imbalance of thiol and disulfide, increased peroxidation of lipids, and decreased antioxidant defense. The occurrence of these changes during symptomatic exacerbation or an active disease state indicates that ROS-related pathways are associated with disease activity. However, most studies relied on measurements of biomarkers in serum, plasma, or erythrocytes, rather than direct measurements of ROS in the nasal mucosa. Therefore, it is unclear whether the observed oxidative imbalance is a cause of AR, a consequence of AR, or an epiphenomenon. Importantly, as demonstrated by the population-based cohort study—although relying on computational exposure modeling (KM-SUB-ELF) that requires further in vivo validation—exposure to ROS during early-life was associated with asthma but not AR. This crucial negative finding suggests that a primary triggering role for exogenous ROS in the pathogenesis of AR has not been definitively established, but rather that endogenous ROS may regulate or amplify a previously established type-2 inflammation. From this perspective, recent studies have focused on the role of ROS produced by inflammatory cells (eosinophils and neutrophils), during the maintenance and amplification of allergic inflammation. Further research on this topic may provide a better understanding the pathophysiological significance of oxidative stress in AR.

### 3.2. Production of ROS by Inflammatory Cells During the Late-Phase of Allergic Inflammation

Mast cell degranulation and histamine release drive the early phase of inflammation in AR, but inflammatory cells, including eosinophils, neutrophils, and T lymphocytes, drive the late-phase, which is characterized by persistent and chronic symptoms [3]. Recent studies have emphasized that these inflammatory cells may directly contribute to the formation of the allergic inflammatory microenvironment by production of cytokines, inflammatory mediators, and ROS [5]. This perspective reinterprets the role of ROS as regulatory factors that connect inflammatory cell activation with tissue responses, rather than as inflammatory byproducts [5,6].

#### 3.2.1. Production of ROS by Eosinophils During Allergic Inflammation

Eosinophils are important effector cells in AR and a major source of ROS during allergic inflammation. Sannohe et al. analyzed the generation of ROS by peripheral eosinophils isolated from patients who had allergic asthma with AR or healthy controls. The results showed that stimulation by a calcium ionophore alone led to greater ROS production by eosinophils in patients than in healthy controls (*p* < 0.05). In addition, the priming effects of two chemokine ligands (eotaxin and Regulated upon Activation, Normal T cell Expressed and Presumably Secreted [RANTES]) were greater in the patients (*p* < 0.05 to 0.01), and these responses were inhibited by an antagonist of the CCR3 receptor. However, the level of CCR3 on the cell surface of eosinophils was similar in the two groups. Application of IL-5 further increased the chemokine-primed production of ROS in normal eosinophils, but this effect was smaller in eosinophils derived from the patients. The authors concluded that this difference was because the eosinophils from patients were already in a primed and activated state [13]. These findings suggest that eosinophils in allergic airway diseases, including AR, may have an activated phenotype and an increased capacity to generate ROS. In particular, excessive ROS production in response to chemokine stimulation indicates that eosinophil-derived ROS are not merely oxidative byproducts, but may contribute to the activation of eosinophils and the persistence of inflammatory responses. However, because this study employed ex vivo experiments using peripheral eosinophils, in vivo validation is needed to confirm the effect of eosinophil-derived ROS within nasal mucosal tissue. Furthermore, it should be noted that the patient cohort in this study included individuals with comorbid allergic asthma, meaning these findings reflect a broader allergic airway phenotype rather than an isolated AR response.

#### 3.2.2. Production of ROS by Neutrophils During the Late-Phase of the Allergic Response

Other studies have proposed that neutrophils generate ROS during late-phase of allergic inflammation. Lavinskiene et al. evaluated changes in neutrophil activation and ROS production after allergen challenge in patients who had AR with allergic asthma and were sensitized to *Dermatophagoides pteronyssinus* (dust mite). The study showed that the IL-8-induced neutrophil chemotaxis was greater in these patients than in healthy controls at baseline (*p* < 0.05), and that this difference persisted after allergen challenge. The serum level of IL-8 in these patients also increased over time (12.0 ± 1.5 pg/mL at baseline, 16.9 ± 1.4 pg/mL at 7 h, 18.1 ± 1.5 pg/mL at 24 h; *p* < 0.05). ROS production by neutrophils was not significantly different at 7 h, but was clearly greater at 24 h. In addition, stimulation by *Staphylococcus aureus* increased ROS production by 15.5 ± 1.4-fold to 19.4 ± 2.1-fold, and simulation by PMA increased ROS production by 125.6 ± 10.8-fold to 175.9 ± 13.1-fold (all *p* < 0.05) [14]. Notably, the increase in ROS was more evident after 24 h than immediately after allergen challenge. This suggests that neutrophil-derived ROS are more closely associated with the late-phase of the inflammatory response. In addition, the simultaneous presence of an increased production of IL-8 and neutrophil activation indicates that ROS generation may not be an isolated phenomenon, but may function as part of a cytokine-driven inflammatory cascade. However, this study was also based on analysis of peripheral neutrophils and the dynamics of ROS within the nasal mucosa may be different. Similar to the aforementioned study, the inclusion of asthma patients in this cohort requires caution when attributing these systemic neutrophil responses exclusively to AR.

#### 3.2.3. Evidence Linking Production of ROS with Eosinophilic Inflammation

Animal experiments support the pathophysiological significance of ROS derived from inflammatory cells during the pathogenesis of AR. Yu et al. evaluated the effects of hydrogen-rich saline (HRS) on oxidative stress and eosinophilic inflammation using an ovalbumin (OVA)-induced guinea pig model of AR. After OVA challenge, the animals showed increased sneezing and scratching behaviors, and had a significantly increased level of serum IgE compared with the control (175.44 ± 10.02 vs. 65.24 ± 8.44 ng/mL, *p* < 0.01). OVA challenge also led to increased levels of ROS in serum and nasal mucosa, accumulation of eosinophils, and expression of eotaxin. HRS treatment significantly decreased the levels of ROS in serum and nasal mucosa (*p* < 0.05), and also suppressed eosinophil accumulation, eosinophil activation, and the levels of eotaxin and IgE. The authors suggested that the decreased level of ROS was related to the attenuation of eosinophilic inflammation [15]. However, it is difficult to attribute the effects of HRS solely to the scavenging of ROS, and these findings alone only provide limited support for ROS as a direct cause of eosinophilic inflammation. Nevertheless, the parallel decrease in ROS and inflammatory responses is consistent with the interpretation that ROS may modulate the intensity and persistence of allergic inflammation.

#### 3.2.4. Pathophysiological Implications of Inflammatory Cell-Derived ROS

Taken together, the evidence to date suggests that eosinophils and neutrophils may be major sources of ROS during the late-phase of the inflammatory response of AR, and that these ROS may activate inflammatory cells and maintain or amplify inflammatory responses. However, most studies measured inflammatory cells from peripheral blood samples or animal models, and there is insufficient evidence from direct measurements of cell-specific generation of ROS by human nasal mucosa. Therefore, although the current evidence is consistent with ROS functioning as a mediator of allergic inflammation, additional tissue-specific human studies are needed to confirm the causal nature and clinical significance of this relationship. Nonetheless, this perspective provides an important basis for understanding pathophysiological mechanisms of ROS at higher levels cell communication that are related to dysfunction of the epithelial barrier, DAMP signaling, and activation of inflammasomes.

### 3.3. Role of ROS in Dysfunction of the Nasal Epithelial Barrier

The nasal epithelium is the first tissue to contacts inhaled allergens, pathogens, and environmental pollutants, but it has other functions beyond acting as a simple physical barrier. Under normal conditions, the tight junctions and the mucociliary clearance system of the nasal epithelium limit the penetration of external stimuli and maintain mucosal homeostasis. This epithelium also regulates the balance between innate and adaptive immune responses by secreting various cytokines, chemokines, and alarmins. Recent studies indicated that dysfunction of the epithelial barrier occurred during the pathophysiology of AR, and additional evidence suggests that this dysfunction may contribute to the increased penetration of allergens and the formation of persistent type 2 inflammation [2,3]. This has led to the view that ROS are not merely markers of oxidative stress but also function as active regulatory factors that connect the integrity of the epithelial barrier with downstream inflammatory signaling.

#### 3.3.1. ROS-Mediated Disruption of the Epithelial Barrier

Shin et al. evaluated the effects of *Alternaria alternata* (a fungal allergen), on the barrier function of the human nasal epithelium [16]. They cultured primary nasal epithelial cells from the inferior turbinate of non-allergic patients using an air–liquid interface culture system. Stimulation of these cells with *Alternaria* increased the level of intracellular ROS (*p* < 0.05) and decreased transepithelial electrical resistance (TEER) (*p* < 0.05). At the same time, there was decreased mRNA and protein expression of ZO-1, occludin, and claudin-1, but no significant change in the level of E-cadherin (an adherens junction marker). These researchers also studied the possible mechanism of this response. Pretreatment with glutathione partially reversed the effect of *Alternaria* on the production of ROS, the decrease in TEER, and the decreased expression of tight junction proteins. Pefabloc (a serine protease inhibitor) had similar protective effects but E-64 (a cysteine protease inhibitor) and pepstatin (an inhibitor of aspartic protease) did not significantly inhibit these changes. The finding that scavenging of ROS and inhibition of serine protease attenuated the dysfunction of the epithelial barrier suggests that allergen-derived serine protease activity may be upstream of ROS generation, and that ROS production may be a key to the disruption of tight junctions [16]. Although this study did not examine patients with AR, it demonstrated that inhaled allergens can induce the generation of ROS in nasal epithelial cells and thereby decrease barrier integrity. Considering that recent studies of AR have emphasized that dysfunction of the epithelial barrier may not only be a consequence of inflammation but may also function in the sensitization phase and in disease initiation [2], ROS-mediated barrier injury may be interpreted as an early pathophysiological response that links the penetration of allergens with subsequent immune activation.

#### 3.3.2. ROS and Epithelial Danger Signaling

ROS function significantly in disrupting the epithelial barrier and modulating danger signaling by promoting the release of epithelium-derived DAMPs in response to PAMPs. This signaling cascade is well exemplified by the Th2 cytokine-DUOX2-ROS-HMGB1 axis, which has been characterized in both primary human nasal epithelial cells from AR patients and an in vivo house dust mite (HDM)-induced AR mouse model [9]. In patients with AR, the level of cytoplasmic HMGB1-positive epithelial cells and the level of HMGB1 in nasal lavage fluid were greater than in normal controls. The in vitro experiments showed that stimulation by IL-4 or IL-13 increased the level of epithelial intracellular ROS and the extracellular release of HMGB1, and these effects were inhibited by a ROS scavenger (N-acetylcysteine, NAC). In addition, down-regulation of DUOX2 (by shRNA) decreased the IL-4/IL-13-induced generation of ROS and the translocation of HMGB1. Their studies of the mouse model showed there was an increased level of ROS in the nasal mucosa, and that an inhibitor of HMGB1 translocation (glycyrrhizic acid) decreased the levels of HDM-specific IgE, eosinophilic inflammation, and goblet cell hyperplasia [9]. HMGB1 is a DAMP molecule, and its extracellular release activates innate immunity and amplifies inflammation. Therefore, these findings indicate that ROS are not merely byproducts of oxidative stress, but may regulate epithelial danger signals and may amplify allergic inflammation through DUOX2-dependent signaling. In particular, the finding that epithelial cells can be an upstream source of a ROS-dependent inflammatory cascade expands the conventional view that eosinophils have a central role in the pathogenesis of AR.

#### 3.3.3. Mitochondrial ROS and Epithelial Dysfunction

Recent studies have proposed mitochondrial ROS (mtROS) functions in AR-associated epithelial dysfunction. Xu et al. analyzed the association between mtROS and the SIRT1/PGC-1α signaling pathway using an OVA-induced AR mouse model and IL-4/IL-13-stimulated human nasal epithelial cells [17]. α-Asarone (ASA) treatment decreased the AR-like symptoms, such as nose rubbing and sneezing, and also decreased the levels of serum OVA-specific IgE and inflammatory mediators (TNF-α, IL-6, and IL-1β). ASA also restored the expression of epithelial barrier-associated proteins (occludin, ZO-1, and E-cadherin), increased the levels of mitochondrial TOM20 and MFN2, and decreased the level of mitochondrial DRP1. The in vitro studies showed that IL-4/IL-13 stimulation increased the levels of cytokines, decreased the TEER, disrupted junctional proteins, and increased the level of mtROS; as above, ASA treatment attenuated these responses. In addition, rotenone weakened the protective effects of ASA, whereas Mito-TEMPO enhanced these effects. Experimental studies using a SIRT1 inhibitor (EX527) and a PGC-1α activator (ZLN005) suggested that these effects may be associated with the SIRT1-dependent mitochondrial pathway of biogenesis [17]. This study extends the conventional perspective centered on ROS generated in the cytosol or by inflammatory cells, and demonstrates that ROS generated in the mitochondria may function directly in disruption of the epithelial barrier and inflammatory amplification. This raises the possibility that nasal epithelial dysfunction in AR is not merely a consequence of the inflammatory response, but is part of the intracellular energy metabolism and stress responses linked to redox imbalance in the mitochondria.

#### 3.3.4. Clinical Significance of ROS-Mediated Epithelial Barrier Dysfunction

These studies of the effect of ROS on the function of the nasal epithelium suggest that ROS may mediate the pathogenesis of AR by regulating the structural stability and immunologic activity of this tissue. ROS are not merely byproducts of cellular damage, but may function as key signaling nodes that connect the dysfunction of the epithelial barrier with immune responses by disruption of tight junctions, release of HMGB1, disruption of mitochondria, and other mechanisms. In particular, ROS may regulate a regulatory axis within the epithelial compartment and may be linked to higher-level pathophysiological pathways, such as activation of inflammasomes, pyroptosis, and mitochondrial stress responses. This perspective suggests the need to move beyond the conventional interpretation of ROS as markers of oxidative stress and to consider them as molecules that connect different structural and functional axes in the pathophysiology of AR. However, most of the current evidence is based on in vitro models and animal experiments, and the effect of different sources of ROS and their clinical significance in human nasal mucosal tissue are uncertain. Therefore, future studies need to identify the spatial and cellular origins of ROS by analysis of human nasal tissues and evaluation of the effect of specific ROS pathways on disease severity and treatment response.

### 3.4. ROS–Inflammasome–IL-1β/Pyroptosis Axis

Recent studies have suggested that ROS may act as upstream regulatory factors that connect the activation of inflammasomes and pyroptosis, which are key axes of innate immune signaling [7,18]. However, rather than regarding this as a single and fully established mechanism, it is more appropriate to interpret it as an integrated mechanistic framework that occurs in various disease models and experimental systems.

The NLRP3 inflammasome is a cytosolic protein complex that is part of the innate immune system which is activated by various ‘danger’ signals. Inflammasome activation increases the activity of caspase-1 and the maturation of IL-1β and IL-18, leading to gasdermin D (GSDMD)-dependent pyroptosis. Previous studies have identified mitochondrial ROS (mtROS) as upstream signals that activate inflammasomes, indicating that inflammasomes link oxidative stress with activation of innate immunity [7,18]. Such ROS-driven signaling may play an important role in the inflammatory amplification phase of AR that occurs after damage of the epithelial barrier, and some evidence suggests that the ROS–inflammasome–pyroptosis axis may function in the immune cells and epithelium of patients with AR.

#### 3.4.1. ROS and NLRP3 Inflammasome Activation in AR

Shi et al. performed a Peripheral Blood Mononuclear Cell (PBMC) -based ex vivo analysis in 45 patients with persistent moderate-to-severe AR and in 23 healthy controls to evaluate the association between inflammasome activation and mitochondrial ROS. The PBMCs from AR patients had greater expression of the NLRP3 and IL-1β mRNAs compared with controls at baseline and following LPS stimulation (all *p* < 0.05). In addition, IL-1β activation was greater in the CD14+ monocyte/macrophage fraction, suggesting that the innate immunity of patients with AR may be in a primed inflammatory state. Their functional analysis demonstrated that LPS priming induced ATP-induced inflammasome activation. They measured mitochondrial ROS in CD14+ cells using a MitoSOX flow cytometry assay and found that the level of mitochondrial ROS after ATP stimulation was higher in the AR group, and that there was a functional association between inflammasome activation and redox imbalance. The mechanistic studies using Mito-TEMPO (an antioxidant that targets mitochondria) showed that this compound significantly decreased ATP-induced secretion of IL-1β (*p* < 0.05). This suggests that mitochondrial ROS are not merely oxidative byproducts but function as upstream regulatory signals that regulate the activity of NLRP3 inflammasomes and the production of IL-1β. Additionally, serum analysis showed that the levels of IL-1β and IL-17 were greater in AR patients (*p* < 0.05), and there was a positive correlation between these two cytokines (r = 0.4073, *p* = 0.0006). Thus, inflammasome-derived IL-1β signaling may be linked to the Th17 axis and contribute to the chronic inflammatory amplification in AR [18].

#### 3.4.2. Environmental ROS Triggers and Epithelial Inflammasome Signaling

Environmental agents can activate the ROS–inflammasome axis in immune cells and the epithelium. Li et al. analyzed the effects of co-exposure to black carbon (BC) and pollen allergen on oxidative stress and inflammasome activation in human nasal epithelial cells (hNECs). These hNECs were derived from the inferior turbinate of 10 non-allergic donors, were cultured using an air–liquid interface culture system, and were then exposed to BC (25 μg/mL), pollen (200 μg/mL), or both agents. BC exposure induced typical oxidative stress responses: production of intracellular ROS, increased lipid peroxidation (MDA), decreased antioxidant capacity (SOD activity), and induction of HO-1. These responses were greater after exposure to both agents. Analysis of inflammasomes also demonstrated that co-exposure led to the greatest level of the NLRP3 protein and secretion of IL-1β. These results indicate that exposure to environmental agents induces the production of ROS and activates inflammasomes in hNECs. Their studies of the underlying mechanism demonstrated that pretreatment with an oxygen scavenger (NAC) inhibited the production of ROS and decreased the levels of NLRP3 and IL-1β. In contrast, an NLRP3 inhibitor (MCC950) and a caspase-1 inhibitor (YVAD) inhibited the maturation of IL-1β but did not affect the generation of ROS. These results suggest that ROS act as upstream activators of inflammasomes and that the ROS-NLRP3 axis may be part of the mechanism by which environmental pollutants cause allergic inflammation of the nasal epithelium [19].

#### 3.4.3. ROS-Dependent Pyroptosis and Damage of the Epithelial Barrier

Yuan et al. analyzed the effects of fine particulate matter (PM2.5) on epithelial injury and pyroptosis in AR by in vivo examination of human nasal mucosa, in vitro examination of HNECs, and examination of an HDM-induced AR mouse model. The nasal mucosa of patients with AR had greater expression of NLRP3, caspase-1, GSDMD, IL-1β, and IL-18 than controls (*p* < 0.05 to *p* < 0.001). This indicates that activation of inflammasomes and pyroptosis occurred at the tissue level. The in vitro studies showed that exposure to PM2.5 induced caspase-1; cleavage of GSDMD; and increased the levels of intracellular ROS, LDH release, and the number of propidium iodide (PI) -positive cells. These changes occurred in a concentration-dependent manner, and were consistent with the typical features of pyroptosis. Their study of the mechanism demonstrated that NAC pretreatment decreased the activation of NLRP3, cleavage of GSDMD, release of IL-1β/IL-18, and generation of ROS. In contrast, AHR knockdown in mice decreased ROS production and pyroptosis, whereas CYP1A1 overexpression had a rescue effect. These findings suggest that PM2.5 induces ROS generation through the AHR/CYP1A1 axis, and that these ROS may damage the epithelial barrier by activating NLRP3 inflammasomes and pyroptosis [20].

#### 3.4.4. The Role of the ROS-Inflammasome Axis in Disease Progression

The studies described here indicate the ROS–inflammasome-IL-1β-pyroptosis axis in AR is not a single and independent pathway. Instead, it is a broad and interconnected pathway that is implicated in amplifying inflammation and connecting the activation of innate immunity with epithelial injury. From the perspective of immune cells, patients with AR have increased NLRP3 inflammasome activity and increased mitochondrial ROS, suggesting that this pathway may function in the maintenance of chronic inflammation by increasing the level of IL-1β, which is correlated with the Th17-associated cytokine IL-17. In the epithelial compartment, exposure to environmental particulates and allergens induces the generation of ROS, activates NLRP3 inflammasomes, and increases the release of IL-1β. The finding that inhibition of ROS production decreases inflammasome signaling suggests that ROS may function as upstream regulators. Finally, pollutants such as PM2.5 can induce ROS-dependent pyroptosis, thereby activating caspase-1 and promoting GSDMD-mediated cell death responses that can contribute to structural damage of the epithelial barrier. Thus, ROS are likely to function as signaling hubs that connect the activation of inflammasomes with pyroptosis. In addition, because ROS may regulate the inflammasome activation/IL-1β maturation/pyroptosis axis, this suggests that ROS in AR may not function solely as a damage-associated byproduct, but rather as a potential inflammatory signal amplification node.

However, this interpretation is mostly based on ex vivo studies of PBMCs, in vitro epithelial models, and animal models. The interactions of different types of cells and their spatial organization within the human nasal mucosa during the pathogenesis of AR have not been sufficiently elucidated. Therefore, future studies need to perform spatial mapping of inflammasome activation at the tissue level with precise determination of the sources of ROS. Nonetheless, the perspective described here suggests that the ROS axis may determine the course of AR progression based on its balance with the endogenous antioxidant defense system. In the next section, we discuss the regulation of this redox balance in AR with a focus on the KEAP1/NRF2 axis.

### 3.5. Mitochondrial ROS and Antioxidant Defense Pathways

Recent studies consider ROS to be part of an active signaling network that is coupled with mitochondrial dysfunction. In particular, mtROS links epithelial injury, activation of inflammasomes, and apoptosis, suggesting that mtROS signaling extends to regulation of specific organelles. In this context, cellular responses such as mitophagy and the KEAP1/NRF2 axis can be understood as compensatory adaptive mechanisms attempting to mitigate ROS-driven inflammatory signaling.

#### 3.5.1. Mitochondrial ROS, Mitophagy, and Epithelial Homeostasis in Allergic Inflammation

Liu et al. analyzed the roles of mitochondrial ‘quality control’ and mtROS production using an OVA-induced AR mouse model and IL-13-stimulated HNECs. In the mouse model, OVA challenge induced the typical AR phenotype: increased sneezing and nasal rubbing, thickening of the epithelium, infiltration of eosinophils, increased serum levels of IgE and Th2 cytokines (IL-4, IL-5, and IL-13). An antioxidant (polydatin, PD) decreased these changes, and high doses markedly decreased tissue inflammation and structural damage. Notably, the effects of PD were not limited simply anti-inflammatory, but also led to the restoration of mitochondrial homeostasis. At the molecular level, PD increased the levels of Pink1, Parkin, LC3B, and Beclin-1 and decreased the level of p62, thereby activating mitophagy. PD also decreased the level of Bax, cleaved caspase-3, and cytochrome c, but increased the level of Bcl-2, indicating suppression of the mitochondrial (intrinsic) apoptosis pathway. Their in vitro experiments demonstrated that IL-13 stimulation also increased the level of mtROS and decreased mitochondrial membrane potential, but that PD improved mitochondrial integrity by decreasing the MitoSOX signal and restoring the JC-1-based membrane potential. This mitochondrial protection decreased the activation of NLRP3 inflammasomes and suppressed IL-1β/IL-18. Moreover, knockdown of Mdivi-1 or Pink1 partially abolished the protective effects of PD. These results suggest that mitophagy is an upstream regulatory step that regulates the production of mtROS and suppresses inflammasomes [21].

#### 3.5.2. ROS-NRF2 Antioxidant Signaling and Regulation of Epithelial Inflammation

The KEAP1/NRF2/HO-1 pathway also regulates ROS responses. Pyun et al. evaluated the role of this antioxidant axis by therapeutic administration of a traditional medicine (*Caesalpinia sappan* Linn. heartwood water extract [CSLW]) to an OVA-induced AR mouse model. CSLW led to decreased infiltration of eosinophils, decreased levels of serum IgE and Th2 cytokines, and decreased sneezing and nasal rubbing. Histological analysis indicated CSLW also attenuated epithelial thickening, goblet cell hyperplasia, and oxidative stress (based on [4-HNE] staining). In the hNEC model, IL-4/IL-13 stimulation increased the secretion of periostin, eotaxin-3, and MUC5AC, and the level of intracellular ROS, and decreased the expression of nuclear NRF2, HO-1, NQO1, and SOD1, indicating suppression of the antioxidant defense system. CSLW treatment decreased the level of ROS, increased the nuclear translocation of NRF2, and upregulated HO-1, NQO1, and SOD1. Interestingly, these changes were not solely attributable to a simple ROS scavenging effect, because there was also decreased ERK phosphorylation, possibly a readjustment of epithelial inflammatory signaling [22]. However, it is important to note that the biological induction of the KEAP1/NRF2/HO-1 axis in such inflammatory environments should be interpreted primarily as a compensatory adaptive response to intense electrophilic or oxidative stress. This molecular induction indicates an active cellular effort to restore redox homeostasis but does not necessarily equate to successful cytoprotection; progressive mucosal damage may still occur if the exogenous or endogenous ROS burden overwhelms this endogenous neutralizing capacity.

#### 3.5.3. Immune-Metabolic Redox Regulation via CD169+ Macrophages and the NRF2 Axis

Qi et al. suggested that CD169+ macrophages simultaneously regulated the redox microenvironment and immune activation in AR based on findings that the level of CD169+ macrophages were increased in the nasal mucosa and nasal lavage fluid of patients with AR compared with controls. In addition, their experiments using an OVA-induced AR mouse model and a CD169-DTR depletion model showed that depletion of CD169+ macrophages decreased nasal symptoms, eosinophilic infiltration, serum IgE, and various cytokines (IL-4, IL-17, IL-23, and IFN-γ). These results indicate that CD169+ macrophages may function as immune network amplification hubs. They also found that under conditions of oxidative stress, CD169+ macrophages increased the expression of NRF2 and HO-1, and this was accompanied by a decreased level of KEAP1 and increased levels of markers of lipid peroxidation (4-HNE and MDA). Their additional results indicated that metabolic–immune coupling promoted dendritic cell activation through alanine metabolism and the SLC38A2-dependent metabolic axis. In particular, SLC38A2 inhibition suppressed dendritic cell maturation and migration, and decreased Th2/Th17-mediated responses. These results show that redox signaling may be coupled with metabolic reprogramming and act as an upstream regulator of immune activation [23].

#### 3.5.4. Interplay Between mtROS and Antioxidant Defense Mechanisms

Taken together, the evidence to date suggests that mtROS and the antioxidant defense system function as a dynamic regulatory network that simultaneously may regulate epithelial integrity and immune activation. At the mitochondrial level, an increased level of mtROS is linked to epithelial apoptosis and inflammasome activation, and PINK1/PARKIN-mediated mitophagy acts as a key protective mechanism that suppresses mitochondrial dysfunction. This indicates that mitochondrial ‘quality control’ functions as an upstream regulatory axis during the pathogenesis of AR. At the transcriptional level, the KEAP1/NEF2/HO-1 axis suppresses the accumulation of ROS and alters epithelial inflammatory signaling, suggesting that the antioxidant response may function as a regulatory switch that controls the extent of immune activation. Finally, at the level of immune metabolites, there is a complex redox–immune coupling in which innate immune cells, such as CD169+ macrophages, form an oxidative stress environment while also activating dendritic cells and adaptive immune responses via several metabolic pathways. These findings suggest that mtROS and antioxidant defense pathways are not independent, but may function as part of a redox balance network that determines the extent of inflammation in AR. Ultimately, the pathophysiology of AR needs to be understood not as a dichotomy of ‘oxidative damage’ versus ‘antioxidant defense,’ but as a dynamic equilibrium in which these two axes interact continuously.

### 3.6. Therapeutic Targets and ROS-Responsive Drug Delivery Strategies

The ROS produced in AR is no longer considered to merely be an oxidative byproduct, but is increasingly recognized as part of an upstream regulatory axis of the inflammatory network underlying this disease. ROS have a much more complex role than previously understood, in that they can activate inflammasomes and induce epithelial injury, but they can also be used as therapeutic targets or triggers for drug delivery systems. This duality explains why current therapeutic approaches are using two different strategies: ROS scavenging and ROS-responsive drug delivery.

#### 3.6.1. Intracellular ROS Scavenging and Modulation of AR Symptoms

Guo et al. conducted a randomized, double-blind, placebo-controlled pilot clinical study to evaluate the effects of intracellular superoxide scavenging on the symptoms of AR. They divided 56 patients with AR into three groups: They divided 56 patients with AR into three groups: one group received TAT-SOD cream applied to the bilateral LI 20 acupoints (TTA group), one group received a vehicle (placebo) cream at the bilateral LI 20 acupoints (placebo group), and one group received the same TAT-SOD cream applied directly to the nasal cavity (TTN group).The main outcome measures were nasal symptom scores and rhinoscopic findings; oxidative stress was also analyzed by measuring serum MDA and endogenous antioxidant enzyme activity (SOD, catalase, and glutathione peroxidase). After two weeks of intervention, the nasal symptom score in the LI20 TAT-SOD group decreased from 6.9 to 3.0, and the overall efficacy rate was 81.0%. In contrast, there was no clear clinical improvement in the placebo group or intranasal TAT-SOD group. An interesting finding was that there were inconsistencies in the changes in biochemical markers and clinical response. In particular, the L120 TAT-SOD group had a decreased level of serum MDA, but no significant change in antioxidant enzyme activity. This suggests that ROS modulation may have acted locally (in a specific tissue environment or signaling context) rather than systemically. In other words, antioxidant intervention may lead to improvements in AR symptoms, but ROS should not be considered at the systemic level, and instead should be considered a spatially restricted signaling system [24].

#### 3.6.2. ROS as a Trigger for Stimulus-Responsive Drug Delivery

In a direction distinct from approaches that directly inhibit the production of ROS or that quench ROS, a novel therapeutic strategy for AR used the ROS in the inflammatory microenvironment as trigger for drug delivery. Zhao et al. developed a ROS-responsive hydrogel loaded with an anticholinergic therapeutic (ipratropium bromide, IB@Gel) and evaluated its potential as an intranasal delivery system. This hydrogel had a TSPBA-PVA structure and was designed to release the therapeutic in a high-ROS environment. The in vivo imaging results showed that the IB@Gel was retained in the nasal mucosa longer than the free drug and it increased local drug exposure through sustained release. Studies of an OVA-induced AR rat model showed that IB@Gel significantly decreased sneezing, nasal rubbing, and nasal secretions, suppressed serum IgE, histamine, and Th2 cytokines (IL-4, IL-5, and IL-13), and decreased eosinophil infiltration and mast cell activation. It also attenuated goblet cell hyperplasia and expression of MUC5AC. These findings show that a ROS-responsive delivery system may function as a context-dependent delivery platform that senses the biochemical state of the inflammatory microenvironment in AR. In other words, therapeutics can use ROS as a target and as endogenous signal that activates a therapeutic system [25].

#### 3.6.3. Clinical Translation of ROS-Targeted and Responsive Therapies

Preliminary proof-of-concept studies that use therapeutic strategies which target ROS as a treatment for AR are exploring two distinct directions. One approach uses an antioxidant intervention that directly decreases the level of intracellular ROS to suppress downstream inflammatory signaling. The second approach uses a stimulus-responsive delivery system in which the ROS-rich environment of AR triggers drug release. Although both approaches target the ROS signaling axis, the first approach directly decreases ROS and the second approach uses ROS as a signal to release a therapeutic. However, only limited and early-stage evidence supports these approaches, and there is a need to better understand the heterogeneity of ROS in the human nasal mucosa, compartment-specific ROS thresholds, and the long-term immunologic effects of these drugs under conditions of chronic inflammation. In addition, the possible adverse effects of these treatments on innate immune signaling, particularly the inflammasome-IL-1β axis and the adaptive immune response, are unclear. Therefore, future studies need to redefine ROS as part of a dynamic signaling node that can change over time and space. The development of stratified therapeutic approaches that consider a patient’s redox status and provide precision intranasal delivery are required.

### 3.7. Limitations and Future Directions

The studies analyzed in this review describe many details regarding the relationship between ROS and AR at the molecular and physiological levels; however, the current literature has several limitations. First, many clinical studies of AR have relied on markers of systemic oxidative stress from serum, plasma, or erythrocyte samples rather than directly measuring ROS within the nasal mucosa. Therefore, it remains difficult to determine whether the reported redox imbalance is a primary driver of nasal inflammation, a downstream consequence, or merely an epiphenomenon. Second, the current evidence encompasses markedly heterogeneous experimental designs, ranging from in vitro human nasal epithelial cell cultures and ex vivo assays to murine OVA- or HDM-induced models and biomaterial engineering platforms. While these diverse systems provide valuable mechanistic insights, they may not fully recapitulate the clinical heterogeneity and pathophysiological complexity of AR in human patients. Synthesizing data across such incommensurable platforms carries an inherent risk of overgeneralization; therefore, the proposed concept of ROS acting as a central “redox regulatory hub” should be interpreted as an evolving conceptual framework rather than a definitively established universal pathway. Importantly, the inclusion of cohorts with comorbid asthma or broader allergic airway diseases in some studies limits the direct generalization of these findings to AR-specific mechanisms. While these mixed-cohort studies provide valuable mechanistic insights into general type 2 airway inflammation, they should not be interpreted at the same evidentiary level as direct experiments utilizing strictly defined, clean AR populations or human nasal-tissue analyses. Third, although robust associations between ROS signaling and inflammatory pathways have been reported, studies establishing strict causality through targeted pharmacologic inhibition, gene knockdown, or rescue experiments remain limited. Fourth, while the concept of “oxidative eustress” is biologically critical for maintaining cellular homeostasis, current AR research is limited by the methodological tools used for ROS detection. The reviewed studies predominantly relied on broad-spectrum fluorophore-based sensors (e.g., DCF/DCFDA) or downstream damage markers. These conventional probes possess well-known technical shortcomings, including susceptibility to auto-oxidation, photobleaching, and a critical lack of specificity for distinct ROS (e.g., the inability to accurately differentiate hydrogen peroxide from superoxide anions). Consequently, these methods cannot reliably distinguish low-level physiological redox signaling from pathological oxidative distress. This methodological limitation inevitably weakens the validity of specific claims in the reviewed literature that attempt to link distinct inflammatory cascades to specific ROS molecules. Finally, the epidemiological evidence discussed in this review relies heavily on computational exposure metrics, such as the KM-SUB-ELF model. While theoretically robust, these models lack direct in vivo validation for estimating the precise individual ROS burden within the nasal mucosa, raising the possibility of exposure misclassification.

Considering these limitations, future research must prioritize the direct quantification of ROS within the human nasal mucosa and integrate these localized measurements with assessments of symptom severity, epithelial barrier integrity, inflammatory cytokine profiles, and environmental allergen exposure. Moreover, rigorous studies are required to accurately distinguish DUOX-derived ROS from mitochondrial ROS (mtROS), evaluate the specific contribution of each source, and validate their spatiotemporal coupling with inflammasome activation, HMGB1 translocation, and the KEAP1/NRF2/HO-1 axis under standardized experimental conditions. Future studies employing highly specific, compartmentalized redox biosensors are urgently needed to accurately measure eustress in the nasal mucosa, and longitudinal epidemiological studies incorporating direct, individualized in vivo biomarker measurements are necessary to confirm modeled associations. Ultimately, establishing the clinical efficacy and safety of ROS modulation or ROS-responsive targeted therapeutic strategies will require well-designed, strictly controlled, and tissue-specific clinical trials.

## 4. Conclusions

The findings of this narrative review suggest that rather than acting as simple etiologic agents, ROS function as context-dependent redox mediators that may regulate inflammatory responses and epithelial integrity within the type 2 inflammatory milieu of AR. While human studies to date have reported alterations in markers of systemic oxidative stress, it remains unclear whether these findings accurately reflect local ROS dynamics at the level of the nasal mucosa. Furthermore, although diverse experimental models have demonstrated strong associations between ROS generation, epithelial barrier dysfunction, and immune cell activation, integrated in vivo evidence linking these heterogeneous findings into a single unifying pathway remains insufficient. Therefore, the role of ROS in AR is more appropriately understood through an evolving conceptual framework as a dynamic redox regulatory network, rather than a definitively established universal mechanism or mere indicators of oxidative damage. Ultimately, rigorous, well-designed clinical studies utilizing tissue-specific measurements of ROS are imperative to bridge this translational gap and fully validate the clinical efficacy of ROS-targeted therapeutic strategies.

## Figures and Tables

**Table 1:** Studies on Reactive Oxygen Species in Allergic Rhinitis.

Author/ Year/ Reference	Study Design	Sample	Detection Method	Target Substance(s) Associated with ROS	Results and Authors’ Conclusions
Min et al., 2021 [9]	Human tissue/fluid analysis + in vitro primary HNE cell mechanistic study + in vivo HDM-induced AR mouse model	Human samples: AR patients n = 20 and controls n = 15. In vitro: primary human nasal epithelial cells cultured at air–liquid interface and stimulated with IL-4 or IL-13. In vivo: HDM-induced AR BALB/c mouse model with or without GA pretreatment	Human nasal mucosa IHC for HMGB1; nasal lavage HMGB1 ELISA; HNE cell Western blot and ELISA for extracellular HMGB1; DCFDA staining for intracellular ROS; DUOX1/DUOX2 Western blot and shRNA knockdown; in vivo nasal ROS imaging using BioNT chemiluminescent nanoprobe; HDM-specific IgE ELISA; PAS and Sirius Red staining	Th2 cytokines IL-4/IL-13; DUOX2; ROS; HMGB1 translocation; NAC as ROS scavenger; GA as HMGB1 translocation inhibitor	AR pts had increased cytoplasmic HMGB1-positive epithelial cells and higher nasal lavage HMGB1 than controls (*p* < 0.05). IL-4/IL-13 increased ROS and extracellular HMGB1 release in primary HNE cells (*p* < 0.05); NAC decreased the ROS elevation and HMGB1 translocation (*p* < 0.05). Th2 cytokines increased DUOX2 expression (*p* < 0.05), and DUOX2 knockdown decreased cytokine-induced ROS generation and HMGB1 translocation (*p* < 0.05). HDM-induced AR mice had greater nasal mucosal ROS than controls (*p* < 0.05), and GA decreased HDM-specific IgE, nasal lavage HMGB1, PAS-positive goblet cell changes, and Sirius Red-positive eosinophilic inflammation (*p* < 0.05). Th2 cytokine-induced DUOX2-dependent ROS generation may promote extracellular translocation of HMGB1 and the development of AR.
Ulusoy et al., 2016 [10]	Human; prospective case–control study	32 patients with seasonal allergic rhinitis (SAR) and 32 age- and sex-matched healthy controls	Blood analysis using a thiol–disulfide homeostasis assay	Native thiol (SH), total thiol (TT), disulfide (SS), SS/SH, SS/TT, and SH/TT ratios	During symptomatic exacerbation, SAR patients had decreased native SH and increased SS compared to asymptomatic periods and controls. The SS/SH and SS/TT ratios increased, while the SH/TT ratio decreased, indicating increased oxidative stress. Symptomatic exacerbation of SAR is associated with an oxidative shift in the thiol–disulfide balance, reflecting increased systemic oxidative stress.
Sadowska-Woda et al., 2010 [11]	Human; prospective controlled clinical study with pre/post-treatment comparison	50 children aged 3–10 years with moderate perennial allergic rhinitis, assessed before and after 2 months of desloratadine 5 mg/day; 11 healthy children as controls	Erythrocyte SOD and catalase activities; erythrocyte MDA by thiobarbituric acid method; plasma hydroperoxides by ferrous oxidation–xylenol orange assay; plasma total antioxidant status by FRAP assay	Oxidative stress markers: MDA and plasma hydroperoxides; antioxidant defense markers: SOD, catalase, total antioxidant status	Untreated perennial AR patients had significantly decreased erythrocyte SOD and catalase activities and increased MDA compared with controls and treated pts (*p* ≤ 0.001). Plasma hydroperoxides were significantly higher in untreated AR pts than in controls; these hydroperoxides decreased in AR pts after desloratadine, but remained above the control level (*p* ≤ 0.001). Total antioxidant status was significantly lower in AR pts than in controls (*p* ≤ 0.001), but the increase after desloratadine was not statistically significant. Oxidative stress is implicated in perennial AR. Desloratadine may have additional in vivo antioxidant effects beyond H1-receptor blockade.
To et al., 2022 [12]	Population-based longitudinal birth cohort study	1284 T-CHEQ Birth Cohort participants in Toronto, born between 1996 and 2000 and followed from birth until outcome, March 31, 2016, or loss to follow-up	Health administrative database linkage; ICD-code-based outcome ascertainment; land-use exposure modeling; KM-SUB-ELF model; Cox proportional hazards regression	Exogenous ROS generated in epithelial lining fluid; PM2.5-related iron Fe and copper Cu; modeled respiratory tract ROS exposure	Modeled ROS exposure at birth was associated with increased risk of childhood asthma (HR 1.11, 95% CI 1.02–1.21, *p* < 0.02), but not AR (HR 0.96, 95% CI 0.88–1.04, *p* = 0.35) or eczema (HR 1.03, 95% CI 0.98–1.09, *p* = 0.24). Fe and Cu individually had no significant association with asthma, AR, or eczema. Early-life exposure to exogenous ROS may increase the risk for childhood asthma, but not AR, in this cohort.
Sannohe et al., 2003 [13]	Human; ex vivo eosinophil functional study comparing allergic patients and healthy controls	15 patients with allergic respiratory disease asthma and/or allergic rhinitis; 11 had allergic rhinitis, 7 had asthma, and 3 had both. 12 healthy nonallergic controls. Peripheral blood eosinophils were isolated for functional assays	Eosinophil isolation by CD16-negative selection; luminol-dependent chemiluminescence after calcium ionophore A23187 stimulation; eotaxin and RANTES priming assays; CCR3 antagonist experiment; flow cytometry for CCR3 expression; IL-5 co-stimulation assays	Eosinophil-derived ROS; eotaxin; RANTES; CCR3; IL-5; calcium ionophore-induced oxidative metabolism	Basal A23187-induced ROS production by eosinophils was greater in AR pts than controls (*p* < 0.05). Eotaxin and RANTES enhanced eosinophil production of ROS, and this priming effect was stronger in AR pts than controls (*p* < 0.05 to *p* < 0.01). A CCR3 antagonist inhibited eotaxin/RANTES-primed ROS production (*p* < 0.05 to *p* < 0.01), but CCR3 expression did not differ between groups. Eosinophils from allergic pts had greater responsiveness to chemokines in oxidative metabolism, suggesting that eotaxin/RANTES/CCR3 signaling may amplify eosinophil-derived ROS in allergic inflammation of airways.
Lavinskiene et al., 2012 [14]	Human; allergen challenge study with ex vivo neutrophil functional assays	47 nonsmoking adults: allergic rhinitis, n = 18; allergic asthma, n = 14; healthy controls, n = 15. AR and asthma patients were sensitized to *Dermatophagoides pteronyssinus*. Peripheral blood was collected 24 h before, 7 h after, and 24 h after bronchial allergen challenge	Bronchial *D. pteronyssinus* challenge; peripheral neutrophil isolation; IL-8-induced chemotaxis assay; FITC-labeled *Staphylococcus aureus* phagocytosis assay; ROS production measured by DHR-123 flow cytometry after PMA or *S. aureus* stimulation; serum IL-8 by ELISA	Neutrophil-derived ROS; PMA-induced oxidative burst; *S. aureus*-induced ROS production; IL-8; late-phase allergen-induced airway inflammation	Peripheral neutrophils in AR pts had increased chemotaxis compared with controls at baseline and after allergen challenge (*p* < 0.05). Serum IL-8 increased after challenge in AR pts from 12.0 ± 1.5 pg/mL (0 h) to 16.9 ± 1.4 pg/mL (7 h), and 18.1 ± 1.5 pg/mL (24 h) (*p* < 0.05). Neutrophil ROS production increased mainly at 24 h after challenge with *S. aureus* (19.4 ± 2.1 vs. 15.5 ± 1.4-fold, *p* < 0.05) and PMA (175.9 ± 13.1 vs. 125.6 ± 10.8-fold, *p* < 0.05). *D. pteronyssinus*-induced late-phase inflammation activates peripheral blood neutrophils in AR and asthma. Increased neutrophil ROS production may reflect systemic inflammatory activation during allergic airway responses.
Yu et al., 2017 [15]	In vivo interventional study using an OVA-induced allergic rhinitis guinea pig model	32 male guinea pigs randomized into 4 groups, n = 8 each: control, normal-HRS, AR-NS, and AR-HRS. AR was induced by OVA sensitization and challenge; HRS was administered intraperitoneally and intranasally for 14 days	Sneezing/scratching frequency; serum IgE and ECP by ELISA; blood eosinophil count; serum ROS by ELISA; nasal mucosal ROS by DCFH-DA fluorescence; serum MDA and SOD assays; nasal mucosal eotaxin by real-time RT-PCR, Western blot, and immunofluorescence	Hydrogen-rich saline HRS; ROS in serum and nasal mucosa; MDA; SOD; eosinophils; ECP; eotaxin	OVA-induced AR increased sneezing/scratching, serum IgE, ROS, MDA, blood eosinophils, ECP, and nasal mucosal eotaxin, but decreased SOD activity. HRS significantly decreased AR symptoms, IgE, ROS, MDA, eosinophil count, ECP, and eotaxin expression, and increased SOD activity (mostly *p* < 0.05 or *p* < 0.01). HRS attenuates allergic inflammation and eosinophil activation in AR, possibly by decreasing ROS-mediated oxidative stress.
Shin et al., 2019 [16]	Ex vivo/in vitro human nasal epithelial cell study using ALI culture	Primary nasal epithelial cells from inferior turbinate mucosa of 10 septal surgery patients without allergy, asthma, aspirin intolerance, or recent corticosteroid/antibiotic use	ALI culture; TER measurement; intracellular ROS fluorescence assay; real-time RT-PCR; Western blot; confocal immunofluorescence microscopy; protease activity assay; heat inactivation; protease inhibitor treatment; glutathione treatment	*Alternaria alternata*; intracellular ROS; serine protease activity; glutathione; tight junction proteins ZO-1, occludin, claudin-1; adherens junction protein E-cadherin	*Alternaria* increased intracellular ROS and decreased TEER in ALI-cultured nasal epithelial cells (*p* < 0.05). It also decreased expression of ZO-1, occludin, and claudin-1 and fluorescence intensity (*p* < 0.05), but did not significantly change E-cadherin. Heat inactivation, glutathione, and a serine protease inhibitor (Pefabloc) attenuated *Alternaria*-induced ROS, TEER decrease, and tight junction loss, but cysteine/aspartic protease inhibitors had little effect. *Alternaria* serine protease induces nasal epithelial barrier dysfunction, at least partly by increasing intracellular ROS production and tight junction disruption.
Xu et al., 2025 [17]	In vivo + in vitro mechanistic study	BALB/c mice with OVA-induced AR treated with ASA 5, 10, or 20 mg/kg; HNEpCs stimulated with IL-4/IL-13 and treated with ASA 25, 50, or 100 μM; mechanistic validation using rotenone, Mito-TEMPO, EX527, ZLN005, and SR-18292	Nasal symptom scoring; H&E staining; NALF total/differential inflammatory cell counts; ELISA for OVA-specific IgE, histamine, TNF-α, IL-6, and IL-1β; CCK-8 assay; TEER measurement; MitoSOX staining for mtROS; Western blot for epithelial barrier and mitochondrial markers; Co-IP for PGC-1α acetylation	ASA; mitochondrial ROS mtROS; SIRT1/PGC-1α pathway; PGC-1α acetylation; epithelial barrier proteins Occludin, ZO-1, E-cadherin; mitochondrial markers TOM20, DRP1, MFN2	In OVA-induced AR mice, ASA decreased sneezing and nose rubbing, serum OVA-specific IgE and histamine, and inflammatory cell infiltration in nasal mucosa and NALF. ASA also decreased TNF-α, IL-6, and IL-1β, restored tight junction-related proteins, and improved mitochondrial status (based on increased TOM20 and MFN2 and decreased DRP1). In IL-4/IL-13–stimulated HNECs, ASA decreased pro-inflammatory cytokines, preserved TEER and epithelial junction protein expression, and suppressed mtROS production. Rotenone decreased the protective effects of ASA, whereas Mito-TEMPO increased the protective effects. ASA increased SIRT1 and PGC-1α activity and promoted PGC-1α deacetylation; these effects were decreased by a SIRT1 inhibitor (EX527) and partly restored by a PGC-1α activator (ZLN005). ASA alleviates AR-associated nasal inflammation, epithelial barrier injury, and mitochondrial dysfunction by suppressing mtROS through SIRT1-dependent PGC-1α deacetylation, supporting the role of mtROS as a mechanistic contributor, rather than a byproduct, of AR inflammation.
Shi et al., 2018 [18]	Human ex vivo mechanistic study using PBMCs from AR patients and healthy controls	45 patients with persistent moderate-to-severe AR and 23 healthy controls; mitochondrial ROS experiments were performed in PBMCs from AR (n = 11) and controls (n = 11)	PBMC isolation; LPS and ATP stimulation; RT-qPCR; flow cytometry; MitoSOX staining; Mito-TEMPO inhibition assay; ELISA; Spearman correlation analysis	Mitochondrial ROS; Mito-TEMPO; NLRP3 inflammasome; IL-1β; IL-17; CD14+ monocyte/macrophage fraction	AR pts had higher basal and LPS-induced IL-1β and NLRP3 expression in PBMCs than controls (*p* < 0.05). CD14+ monocytes/macrophages from AR pts also had greater IL-1β activation after LPS stimulation (*p* < 0.05). In LPS-primed PBMCs, ATP-induced mitochondrial ROS was higher in AR than controls, and Mito-TEMPO decreased the ATP-induced IL-1β secretion (*p* < 0.05). Serum IL-1β and IL-17 were increased in AR pts (*p* < 0.05) and positively correlated (r = 0.4073, *p* = 0.0006). MtROS may promote IL-1β production through NLRP3 inflammasome activation in PBMCs from AR pts, potentially linking systemic innate immune activation with IL-17-related inflammation.
Li et al., 2020 [19]	Ex vivo human nasal epithelial cell study using ALI culture and pollutant/allergen exposure	Inferior turbinate mucosa from 10 non-allergic patients undergoing nasal surgery; hNECs cultured at air–liquid interface and exposed to BC 25 μg/mL, pollen 200 μg/mL, or BC + pollen for 24 h	ALI-hNEC culture; CCK-8 assay; DCFDA ROS assay; MDA assay; SOD activity assay; qRT-PCR; ELISA; Western blot; immunofluorescence staining; NAC, MCC950, and YVAD inhibition assays	BC; pollen allergen; ROS; MDA; SOD; HO-1; NLRP3 inflammasome; caspase-1; IL-1β; NAC	BC increased intracellular ROS, MDA, HO-1, and IL-1β and decreased SOD activity; pollen alone had little effect, but BC + pollen amplified oxidative stress and IL-1β production. BC + pollen increased NLRP3 expression and IL-1β secretion. NAC decreased ROS, MDA, HO-1, NLRP3, and IL-1β (*p* < 0.01). MCC950 and YVAD decreased IL-1β (*p* < 0.01) without decreasing the levels of ROS or NLRP3. Exposure to BC, especially with pollen, promotes nasal epithelial inflammation through ROS-dependent NLRP3-caspase-1-IL-1β signaling.
Yuan et al., 2025 [20]	Human tissue analysis + in vitro HNEC mechanistic study + in vivo HDM-induced AR mouse model	Nasal mucosa from AR patients and controls; HNECs exposed to PM2.5; HDM-induced AR mouse model	LDH release assay; propidium iodide (PI) staining; NAC pretreatment; AHR knockdown; CYP1A1 overexpression	PM2.5; NLRP3 inflammasome; caspase-1; GSDMD (pyroptosis); IL-1β; IL-18; AHR/CYP1A1 axis	Nasal mucosa of AR patients had greater expression of NLRP3, caspase-1, GSDMD, IL-1β, and IL-18. In vitro, PM2.5 increased caspase-1, GSDMD cleavage, intracellular ROS, and LDH release. NAC decreased ROS, NLRP3 activation, and GSDMD cleavage. AHR knockdown decreased ROS and pyroptosis, while CYP1A1 overexpression reversed this. PM2.5 induces ROS generation through the AHR/CYP1A1 axis, which damages the epithelial barrier by activating NLRP3 inflammasomes and pyroptosis.
Liu et al., 2023 [21]	In vivo + in vitro mechanistic study	Female BALB/c mice with OVA-induced AR treated with polydatin PD 30 or 45 mg/kg, n = 8 per group; HNEpCs stimulated with IL-13 10 ng/mL and treated with PD 100 or 200 μM; mechanistic validation using Mdivi-1 and PINK1 siRNA	Nasal symptom scoring; H&E staining; ELISA; flow cytometry; TUNEL assay; MitoSOX for mtROS; JC-1 for mitochondrial membrane potential; immunofluorescence and Western blot	PD; mitochondrial ROS mtROS; PINK1-Parkin-mediated mitophagy; mitochondrial membrane potential; NLRP3 inflammasome; apoptosis markers Bax, Bcl-2, cleaved caspase-3, cytochrome C	In OVA-induced AR mice, PD decreased sneezing/rubbing, epithelial thickening, eosinophil infiltration, serum IgE, NALF eosinophils, IL-4, IL-5, IL-13, and partially restored the Th1/Th2 balance (mostly *p* < 0.01). PD increased PINK1, PARKIN, LC3B, and BECLIN and decreased P62 and apoptosis-related changes in nasal tissue. In IL-13–stimulated HNECs, PD decreased mtROS, improved mitochondrial membrane potential, increased PINK1/Parkin-related mitophagy markers, and suppressed NLRP3 inflammasome and apoptosis proteins (mostly *p* < 0.01). Mdivi-1 treatment or PINK1 knockdown attenuated PD-associated protection. PD may alleviate AR by promoting PINK1-Parkin-mediated mitophagy, thereby decreasing mtROS production, NLRP3 inflammasome activation, mitochondrial damage, and epithelial apoptosis.
Pyun et al., 2022 [22]	In vivo + in vitro mechanistic study using an OVA-induced AR mouse model and IL-4/IL-13–stimulated primary HNEpCs	Female BALB/c mice with OVA-induced AR treated orally with CSLW 30 or 100 mg/kg or dexamethasone 1 mg/kg; primary HNEpCs stimulated with IL-4/IL-13 and treated with CSLW 1, 3, or 10 μg/mL or NAC	UPLC-MS/MS profiling of CSLW; nasal symptom scoring; NALF total cell/eosinophil counts; serum OVA-specific IgE, histamine, IL-5, IL-13 by ELISA; H&E, PAS, Giemsa, IHC for periostin, MUC5AC, 4-HNE; HNEpC ELISA for eotaxin-3, periostin, MUC5AC; CellROX staining for intracellular ROS; Western blot for p-ERK, Keap1, HO-1, NQO1, SOD1, and Nrf2	CSLW; intracellular ROS; 4-HNE; Keap1/Nrf2/HO-1 pathway; NQO1; SOD1; ERK-MAPK; eotaxin-3; periostin; MUC5AC	In OVA-induced AR mice, CSLW decreased sneezing/rubbing, NALF total cells and eosinophils, serum OVA-specific IgE, histamine, IL-5, and IL-13, and increased nasal epithelial thickening, goblet cell hyperplasia, eosinophil infiltration, periostin, and expression of MUC5AC and 4-HNE (mainly *p* < 0.05 to *p* < 0.001 vs. AR group, depending on dose and endpoint). In IL-4/IL-13–stimulated HNECs, CSLW decreased eotaxin-3, periostin, MUC5AC, and intracellular ROS, inhibited ERK phosphorylation, decreased KEAP1, and increased HO-1, NQO1, SOD1, and nuclear NRF2 (mostly *p* < 0.05 to *p* < 0.001). CSLW may attenuate allergic nasal inflammation by suppressing ROS-related epithelial inflammatory responses and regulating the ERK-MAPK and Keap1/Nrf2/HO-1 antioxidant pathway.
Qi et al., 2024 [23]	Human nasal mucosa analysis + OVA-induced AR mouse model using CD169-DTR macrophage depletion + in vitro CD169+ macrophage/DC co-culture + metabolomics	Human nasal mucosa/nasal lavage samples from AR and non-AR controls; OVA-induced AR mice with or without CD169+ macrophage depletion; flow-sorted mouse CD169+ macrophages; dendritic cell co-culture system	Immunofluorescence staining; flow cytometry; H&E staining; ELISA; untargeted metabolomics; UHPLC-Q-TOF MS; qPCR; Western blot; alanine assay; scratch assay; Transwell assay; co-culture assay	CD169+ macrophages; Keap1/Nrf2/HO-1 axis; MDA; 4-HNE; alanine; SLC38A2; dendritic cell maturation/migration	CD169+ macrophages were increased in AR nasal mucosa and nasal lavage fluid (*p* < 0.001). CD169+ macrophage depletion decreased AR-like behaviors, nasal eosinophils, IgE, IL-4, IL-17, IL-23, IFN-γ, Th2/Th17 cells, and dendritic cell maturation (mostly *p* < 0.05 to *p* < 0.001). Metabolomics suggested changes in alanine-related pathways. In isolated CD169+ macrophages, LPS altered Keap1/Nrf2/HO-1 markers and increased oxidative stress-related products (MDA and 4-HNE) and alanine (*p* < 0.001). CD169+ macrophages may promote immune responses in AR through alanine metabolism, dendritic cell activation, and oxidative stress-related Keap1/Nrf2/HO-1 signaling. There was no direct ROS quantification, so the ROS-related mechanism was only based on measurements of markers/pathways.
Guo et al., 2016 [24]	Human; randomized, double-blind, placebo-controlled pilot clinical study	56 AR patients: TAT-SOD at LI 20 acupoints, n = 21; placebo at LI 20, n = 17; TAT-SOD directly to nasal cavity, n = 18	Nasal symptom score; rhinoscopy; serum MDA; serum SOD, CAT, and GPx colorimetric assays	Intracellular superoxide; TAT-SOD; MDA; SOD, CAT, GPx	After 2 weeks, TAT-SOD applied to LI 20 decreased total nasal symptom score from 6.9 to 3.0 (placebo: 7.1 to 6.7; direct nasal TAT-SOD: 7.1 to 6.9). The LI 20 TAT-SOD group had an overall efficacy rate of 81.0%, with rhinoscopic improvement of nasal edema in 11 pts. Placebo and direct nasal application led to minimal or no improvement. Serum MDA decreased only in the LI 20 TAT-SOD group (10.8 to 9.0 nmol/L); it increased in the placebo and direct nasal TAT-SOD groups. Serum levels of SOD, CAT, and GPx activities were not significantly changed. Local intracellular superoxide scavenging may alleviate AR symptoms and decrease lipid peroxidation. However, the proposed acupoint-related ROS transport mechanism remains unproven because nasal mucosal ROS and direct intercellular superoxide transport were not directly measured.
Zhao et al., 2024 [25]	In vitro hydrogel characterization + in vivo OVA-induced AR rat treatment study	HNEpCs for cytocompatibility; male SD rats with OVA-induced AR, randomized into control, AR, IB, Gel, and IB@Gel groups, n = 6 per treatment group	Hydrogel morphology by SEM; ROS responsiveness using 1 mM H_2_O_2_; in vitro IB release by HPLC; in vivo nasal retention using Cy5 fluorescence imaging and CLSM; AR symptom scoring; nasal secretion weight; ELISA	ROS-responsive hydrogel; H_2_O_2_-triggered gel degradation; IB; MUC5AC; OVA-IgE; histamine; Th2 cytokines IL-4, IL-5, IL-13	H_2_O_2_ degraded the hydrogel and accelerated IB release under ROS-like conditions. In OVA-induced AR rats, IB@Gel had prolonged nasal retention (up to 24 h) and led to decreases in nose scratching, sneezing, nasal secretion, OVA-IgE, histamine, IL-4, IL-5, IL-13, inflammatory cell infiltration, mucus secretion, and MUC5AC expression (mostly *p* < 0.05 to *p* < 0.0001). AR-associated high ROS can be used to trigger sustained intranasal drug release. IB@Gel improves AR symptoms mainly by decreasing allergic inflammation and MUC5AC-mediated mucus hypersecretion.

## Data Availability

No new data were created or analyzed in this study. Data sharing is not applicable to this article.

## Abbreviations

4-HNE, 4-hydroxynonenal; A23187, calcium ionophore A23187; ALI, air–liquid interface; AR, allergic rhinitis; ASA, alpha-asarone (α-asarone); ATP, adenosine triphosphate; BALB/c, inbred laboratory mouse strain; Bax, BCL2-associated X protein; Bcl-2, B-cell lymphoma 2; BC, black carbon; BioNT, chemiluminescent reactive-oxygen-species-detection nanoprobe (commercial reagent); CAT, catalase; CCK-8, Cell Counting Kit-8; CCR3, C-C chemokine receptor type 3; CD14, cluster of differentiation 14; CD16, cluster of differentiation 16; CI, confidence interval; CLSM, confocal laser scanning microscopy; Co-IP, co-immunoprecipitation; CSLW, *Caesalpinia sappan* Linn. water extract; Cu, copper; Cy5, cyanine 5 (fluorescent dye); DCFDA, 2′,7′-dichlorodihydrofluorescein diacetate; DCFH-DA, 2′,7′-dichlorodihydrofluorescein diacetate; DHR-123, dihydrorhodamine 123; DRP1, dynamin-related protein 1; DTR, diphtheria toxin receptor; DUOX1, dual oxidase 1; DUOX2, dual oxidase 2; ECP, eosinophil cationic protein; ELISA, enzyme-linked immunosorbent assay; ERK, extracellular signal-regulated kinase; EX527, SIRT1 inhibitor (selisistat); Fe, iron; FITC, fluorescein isothiocyanate; FRAP, ferric reducing antioxidant power; GA, glycyrrhizic acid; GPx, glutathione peroxidase; H&E, hematoxylin and eosin; H2O2, hydrogen peroxide; HDM, house dust mite; hNEC, human nasal epithelial cell; HMGB1, high mobility group box 1; HNEpC, human nasal epithelial cell; HO-1, heme oxygenase-1; HPLC, high-performance liquid chromatography; HR, hazard ratio; HRS, hydrogen-rich saline; IB, ipratropium bromide; ICD, International Classification of Diseases; IFN-γ, interferon gamma; IgE, immunoglobulin E; IHC, immunohistochemistry; IL-1β, interleukin-1 beta; IL-4, interleukin-4; IL-5, interleukin-5; IL-6, interleukin-6; IL-8, interleukin-8; IL-13, interleukin-13; IL-17, interleukin-17; IL-23, interleukin-23; IQR, interquartile range; JC-1, mitochondrial-membrane-potential dye (5,5′,6,6′-tetrachloro-1,1′,3,3′-tetraethylbenzimidazolylcarbocyanine iodide); Keap1, Kelch-like ECH-associated protein 1; KM-SUB-ELF, kinetic multi-layer model of surface and bulk chemistry in the epithelial lining fluid; LC3B, microtubule-associated protein 1A/1B light chain 3B; LI 20, Large Intestine 20 (acupoint); LPS, lipopolysaccharide; MAPK, mitogen-activated protein kinase; MCC950, NLRP3 inflammasome inhibitor; MDA, malondialdehyde; Mdivi-1, mitochondrial division inhibitor 1; MFN2, mitofusin 2; Mito-TEMPO, mitochondria-targeted antioxidant; MitoSOX, mitochondrial superoxide indicator dye; MUC5AC, mucin 5AC; NAC, N-acetylcysteine; NALF, nasal lavage fluid; NLRP3, NLR family pyrin domain-containing 3; NQO1, NAD(P)H quinone dehydrogenase 1; Nrf2, nuclear factor erythroid 2-related factor 2; OVA, ovalbumin; PAS, periodic acid–Schiff (stain); PBMC, peripheral blood mononuclear cell; PD, polydatin; Pefabloc, serine protease inhibitor (4-(2-aminoethyl)benzenesulfonyl fluoride, AEBSF); p-ERK, phosphorylated extracellular signal-regulated kinase; PGC-1α, peroxisome proliferator-activated receptor gamma coactivator 1-alpha; PINK1, PTEN-induced kinase 1; PMA, phorbol 12-myristate 13-acetate; P62, sequestosome-1 (SQSTM1); qPCR, quantitative polymerase chain reaction; qRT-PCR, quantitative reverse transcription polymerase chain reaction; RANTES, regulated on activation, normal T cell expressed and secreted (CCL5); ROS, reactive oxygen species; RT-PCR, reverse transcription polymerase chain reaction; RT-qPCR, reverse transcription quantitative polymerase chain reaction; SAR, seasonal allergic rhinitis; SD, Sprague–Dawley (rat strain); SEM, scanning electron microscopy; SH, native thiol; shRNA, short hairpin RNA; SIRT1, sirtuin 1; siRNA, small interfering RNA; SLC38A2, solute carrier family 38 member 2; SOD, superoxide dismutase; SOD1, superoxide dismutase 1; SR-18292, PGC-1α inhibitor; SS, disulfide; T-CHEQ, Toronto Child Health Evaluation Questionnaire (birth cohort); TAT-SOD, trans-activator of transcription–superoxide dismutase fusion protein; TEER, transepithelial electrical resistance; TER, transepithelial resistance; TNF-α, tumor necrosis factor alpha; TOM20, translocase of outer mitochondrial membrane 20; TT, total thiol; TUNEL, terminal deoxynucleotidyl transferase dUTP nick-end labeling; UHPLC-Q-TOF MS, ultra-high-performance liquid chromatography quadrupole time-of-flight mass spectrometry; UPLC-MS/MS, ultra-performance liquid chromatography–tandem mass spectrometry; YVAD, caspase-1 inhibitor (Ac-YVAD-CMK); ZLN005, PGC-1α activator; ZO-1, zonula occludens-1.
